# Senataxin Attenuates DNA Damage Response Activation and Suppresses Senescence

**DOI:** 10.3390/antiox13111337

**Published:** 2024-10-31

**Authors:** Mingyang Li, Genbao Shao

**Affiliations:** Department of Basic Medicine, School of Medicine, Jiangsu University, Zhenjiang 212013, China; limy@ujs.edu.cn

**Keywords:** oxidative stress, senataxin, SETX, DNA double-strand break repair, senescence, DNA damage response, apoptosis

## Abstract

Oxidative stress, driven by reactive oxygen species (ROS) such as hydrogen peroxide (H_2_O_2_), induces DNA double-strand breaks (DSBs) that compromise genomic integrity. The DNA Damage Response (DDR), primarily mediated by ATM and ATR kinases, is crucial for recognizing and repairing DSBs. Senataxin (SETX), a DNA/RNA helicase, is critical in resolving R-loops, with mutations in *SETX* associated with neurodegenerative diseases. This study uncovers a novel function of senataxin in modulating DDR and its impact on cellular senescence. Senataxin is shown to be crucial not only for DSB repair but also for determining cell fate under oxidative stress. *SETX* knockout cells show impaired DSB repair and prolonged ATM/ATR signaling detected by Western blotting, leading to increased senescence, as indicated by elevated β-galactosidase activity following H_2_O_2_ exposure and I-PpoI-induced DSBs. Wild-type cells exhibit higher apoptosis levels compared to *SETX* knockout cells under H_2_O_2_ treatment, suggesting that senataxin promotes apoptosis over senescence in oxidative stress. This indicates that senataxin plays a protective role against the accumulation of senescent cells, potentially mitigating age-related cellular decline and neurodegenerative disease progression. These findings highlight senataxin as a critical mediator in DDR pathways and a potential therapeutic target for conditions where cellular senescence contributes to disease pathology.

## 1. Introduction

Oxidative stress arises from an imbalance between the production of reactive oxygen species (ROS) and the cellular antioxidant defenses, leading to potential damage to various biomolecules, including DNA, proteins, and lipids [1]. Common ROS include superoxide anion, hydrogen peroxide (H_2_O_2_), and hydroxyl radicals [2]. These reactive molecules are generated as natural byproducts of cellular metabolism, particularly within the mitochondria, but their levels can increase dramatically under conditions of environmental stress, such as UV radiation, pollution, and inflammation [3]. Persistent oxidative stress can disrupt cellular function and contribute to the pathogenesis of numerous diseases, including cancer, cardiovascular diseases, and neurodegenerative disorders [4,5].

DNA double-strand breaks (DSBs) are one of the major deleterious types of DNA lesions that threaten genomic integrity and cell viability. DSBs spontaneously arise from replication fork collapse occurring at unrepaired lesions, such as those induced by reactive oxygen species of natural oxidative metabolism [6]. DSBs also occur by exposure to exogenous DNA damaging agents, including ionizing radiation and radiomimetic chemicals [7]. Additionally, DSBs produced by endogenous nucleases are required for various programmed genome recombination events, such as meiosis [8], mating-type switching in yeast [9], V(D)J recombination [10], and immunoglobulin class switching [11] during lymphocyte development. The formed DSBs, wherein two complementary strands of DNA duplex are broken at sufficiently close sites, are repaired by two different pathways: either error-free homologous recombination or error-prone non-homologous end joining [12]. Loss-of-function mutations in many factors participating in the DSB signaling and repair pathway can result in failure to repair DSBs [13]. This failure has detrimental consequences, including increased mutations promoting carcinogenesis [14] and premature aging [15].

The DNA Damage Response (DDR) is crucial for preserving genomic integrity by detecting and repairing DNA lesions [16,17]. Central to this process are the ATM and ATR kinases, which are activated by DSBs and replication stress, respectively [18,19]. ATM, which rapidly localizes to DSB sites, is primarily responsible for DSB repair, particularly through homologous recombination [20]. In contrast, ATR responds to a broader spectrum of DNA lesions, including those affecting replication, and facilitates nucleotide excision repair (NER) to address bulky DNA damage, such as UVC-induced cyclobutane pyrimidine dimers (CPDs) [21]. Activation of ATM and ATR kinases results in the phosphorylation of critical substrates, including Chk1, Chk2, and p53, which are essential for maintaining genome stability and cellular viability under genotoxic stress [22]. These phosphorylation cascades coordinate key cellular responses, such as cell cycle arrest, DNA repair, apoptosis, and senescence, thereby preventing genetic mutations and ensuring cellular homeostasis [23,24,25].

Cellular senescence is a state of irreversible cell cycle arrest that serves as a crucial defense against the proliferation of damaged cells [26], significantly influencing aging and age-related diseases [27,28]. A key driver of senescence is the accumulation of oxidative damage [29], leading to genomic instability and the formation of DNA DSBs [30,31]. These breaks are particularly hazardous as they can cause chromosomal aberrations if not properly repaired [32]. However, the efficiency of DSB repair declines with age, resulting in an increase in unrepaired DNA damage [33]. This accumulation of oxidative damage and persistent DSBs accelerates cellular senescence and contributes to premature aging [34,35,36], highlighting the importance of understanding and potentially targeting DSB repair mechanisms to mitigate age-related decline and improve healthspan.

Senataxin, a protein encoded by the *SETX* gene, is integral to maintaining genomic stability through its involvement in DNA repair processes [37,38]. As a member of the DNA/RNA helicase family, senataxin is critical in resolving R-loops—structures formed during transcription that consist of a DNA–RNA hybrid and a displaced single-stranded DNA [39,40]. These R-loops, if unresolved, can lead to genomic instability, replication stress, and increased susceptibility to DNA damage [41]. Senataxin’s helicase activity facilitates the unwinding of these structures, thus preventing their harmful accumulation [42]. Additionally, senataxin is involved in the repair of DSBs, a particularly lethal form of DNA damage [43,44]. Mutations in the *SETX* gene have been linked to neurodegenerative diseases, such as amyotrophic lateral sclerosis (ALS4) and ataxia with oculomotor apraxia type 2 (AOA2), underscoring the importance of senataxin in both neuronal health and genomic integrity [45,46,47].

This study uncovers a novel role of senataxin in modulating cellular senescence within the DDR pathway, particularly in the context of oxidative stress and DSB repair. *SETX* knockout cells showed decreased sensitivity to H_2_O_2_-induced apoptosis, impaired DSB repair kinetics with prolonged ATM/ATR signaling, and elevated senescence as indicated by elevated β-galactosidase activity. A key finding is that senataxin promotes apoptosis over senescence in response to H_2_O_2_ treatment, revealing its protective role in preventing premature cellular aging. Additionally, senataxin was found to protect cells from UVC-induced apoptosis but did not significantly affect NER of UVC-induced CPDs. These results position senataxin as a critical factor in balancing apoptosis and senescence, particularly under oxidative stress, underscoring its broader role in maintaining genomic stability and preventing age-related cellular decline.

## 2. Materials and Methods

### 2.1. Cell Lines and Culture

Immortalized human fibroblast R2F cells [48] were cultured in DMEM/F12 (Gibco, Grand Island, NY, USA) supplemented with 15% newborn calf serum, 10 ng/mL epidermal growth factor (PeproTech, Cranbury, NJ, USA), 100 U/mL penicillin, and 100 μg/mL streptomycin (Gibco). Cell cultures were maintained in a humidified atmosphere of 5% CO_2_ at 37. For the establishment of cell lines with the inducible expression of I-PpoI endonuclease, cells were transduced using a lentivirus packaged with the lentiviral construct pLenti-HA-I-PpoI-BSD for 4 days. Stable transduced cell lines were generated by selection with 10 μg/mL blasticidin for one week.

### 2.2. Compounds and Antibodies

The following reagents were used: antibodies: anti-senataxin (Novus Biologicals, Littleton, CO, USA, NBP1-94712), anti-Rpb1 (Cell Signaling, Danvers, MA, USA, #2629), anti-phospho-ATM (Ser1981) (Cell Signaling, #4526), anti-ATM (Santa Cruz, Dallas, TX, USA, sc-377293), anti-phospho-ATR (Thr1989) (GeneTex, Irvine, CA, USA, GTX128145), anti-ATR (Santa Cruz, sc-515173), anti-phospho-Chk2 (Thr68) (Cell Signaling, #2661), anti-phospho-Chk1 (Ser345) (Cell Signaling, #2341), anti-phospho-p53 (Ser15) (Cell Signaling, #9284), anti-p53 (Cell Signaling, #2524), anti-HA tag (Santa Cruz, sc-7392), and anti-GAPDH (Santa Cruz, sc-47724).

### 2.3. Plasmid Constructs

For constructing the plasmid pLentiCRISPR-sgSETX-GFP-loxP, the Cre recombinase-recognized loxP site was PCR amplified and cloned into the 3′-LTR of pLentiCRISPR v2 (Addgene, Watertown, MA, USA, 52961). Then the fragment containing GFP was generated by PCR from pmaxGFP (Lonza, Basel, Switzerland) and cloned into pLentiCRISPR-loxP to replace the puromycin resistance gene. The synthesized sequence of *SETX* targeting sgRNA designed by the CRISPR design tool CRISPick (https://portals.broadinstitute.org/gppx/crispick/public, accessed on 26 May 2023) was inserted into pLentiCRISPR-GFP-loxP. For con-structing the plasmid pLenti-DD-HA-ER-I-PpoI, the coding sequence of DD-HA-ER-I-PpoI generated by PCR amplification from pCL20C-ddIPpoI (Addgene, 49053) was cloned into a lentiviral vector with a blasticidin resistance gene (BSD). All the sequences were verified by Sanger sequencing.

### 2.4. CRISPR/Cas9 Knockout of Human SETX

*SETX* knockout was created in R2F cells. Exon 3 of *SETX* was targeted for CRISPR/Cas9 cleavage. Cells were transduced with the lentiviral construct pLentiCRISPR-sgSETX-GFP-loxP. Five days after transduction, GFP-positive cells were seeded as single cells per well in a 384-well plate by serial dilution. Individual cell clones were expanded when they reached confluence. Sanger sequencing was carried out on the CRISPR/Cas9-targeted region amplified from genomic DNA. Two knockout candidates with frameshift insertion-deletions in both alleles were validated by immunoblotting. The U3 region containing a single loxP site of the 3′-LTR of pLentiCRISPR-sgSETX-GFP-loxP was duplicated into the 5′-LTR during reverse transcription of viral RNA after cell transduction. The GFP expression cassette flanked by two loxP sites in knockout cells was removed through Cre-mediated recombination after transduction with the lentiviral construct pLM-CMV-R-Cre (Addgene, 27546) for one week.

### 2.5. Cell Viability Assay

Cells were collected after specific treatments and resuspended in PBS. The cell suspension was mixed with 0.4% trypan blue solution and incubated for 3 min at room temperature. For each sample, stained and unstained cells were counted in a hemacytometer. The percentage of viable cells was calculated.

### 2.6. Apoptosis Detection Assay

The induction of apoptosis was detected using the Dead Cell Apoptosis Kit with Alexa Fluor 488 Annexin V and PI (ThermoFisher, Waltham, MA, USA, V13241) according to the manufacturer’s instructions. After the apoptosis induction period, cells from a 6-well plate were harvested and gently washed with ice-cold PBS. Cells were then resuspended in 1× binding buffer supplemented with Annexin V-Alex Fluor 488 and PI, followed by incubation at room temperature for 15 min. Fluorescence of the stained cells was measured by flow cytometry.

### 2.7. Senescence-Associated β-Galactosidase (SA-β-gal) Assay

Cell senescence was detected using the Senescence β-Galactosidase Staining Kit (Cell Signaling, #9860), according to the manufacturer’s protocol. In brief, treated cells were rinsed with PBS and fixed with a fixative solution at room temperature for 15 min. Fix cells were washed with PBS and then incubated with fresh β-Galactosidase Staining Solution containing X-gal at 37 °C overnight. Images were taken under a light microscope. The SA-β-gal staining positive cells were analyzed and quantified using ImageJ software, version 1.54f.

### 2.8. Double-Strand Break Repair Assay

Cells were infected with lentiviruses encoding pLenti-DD-HA-ER-I-PpoI. After 4 days of transduction, cells stably expressing the I-PpoI fusion protein (DD-HA-ER-I-PpoI) were selected by maintaining them with 10 μg/mL blasticidin for one week. The selected cells were treated with 1 μM Shield-1 and 2 μM 4-hydroxytamoxifen (4-OHT) to generate I-PpoI-induced DNA DSBs. After 5 h treatment, cells were washed with PBS and incubated with fresh complete medium to repair DSBs at 37 °C. Genomic DNA was extracted from cells at the indicated repair time points. I-PpoI-induced DSBs at the 28S rDNA locus were subjected to analysis by quantitative PCR. Briefly, genomic DNA was denatured and annealed with 28S-T. Primer extension was performed. The reaction product was used for qPCR with the primer pair of 28S-F and 28S-R. The result of qPCR reflects the original amount of DSBs at the 28S rDNA region.

### 2.9. Protein Extraction and Immunoblotting Analysis

After treatment, cells were washed twice with ice-cold PBS, harvested, and lysed by vortexing with a phenol solution (equilibrated with 10 mM Tris HCl, pH 8.0, with 1 mM EDTA, Sigma, St. Louis, MO, USA) containing β-mercaptoethanol (Fisher) for 15 min. Phenol-extracted proteins were precipitated by adding methanol containing 0.1 M ammonium acetate. The pelleted proteins were washed once with methanol containing 0.1M ammonium acetate, and twice with ice-cold 80% acetone. The air-dried pellet was dissolved in SDS-PAGE loading buffer (120 mM Tris-Cl, 20% glycerol, 4% SDS, 0.2% bromophenol blue, pH 6.8) supplemented with 5% β-mercaptoethanol, and boiled for 5 min. Protein concentration was measured using a Qubit fluorometer (Invitrogen, Carlsbad, CA, USA). A total of 10 µg of protein extract per well was separated by sodium dodecyl sulfate-polyacrylamide gel electrophoresis (SDS-PAGE) and blotted onto polyvinylidene fluoride (PVDF) membranes (Millipore, Burlington, MA, USA). Membranes were washed and hybridized with indicated primary antibodies diluted in TBST containing 1% fish gelatin at 4 °C overnight. Then membranes were washed and probed with horseradish peroxidase-conjugated secondary antibodies (Invitrogen). Using a chemiluminescence detector (ChemiDoc XRS+ Imaging System, Bio-Rad, Hercules, CA, USA) and ECL reagent (SuperSignal™ West Femto Maximum Sensitivity Substrate, Thermo Scientific), the protein of interest was detected. The relative intensity of the immunoblotting bands was measured using ImageJ.

### 2.10. UV-Induced CPDs Analysis

Cells cultured to the late log phase were irradiated with 25 J/m^2^ of UVC (wavelength 254 nm). Fresh complete medium was added to the irradiated cells, and the cells were incubated at 37 °C. Cells were harvested at the indicated repair incubation times, and then genomic DNA was isolated. Southern blot was applied to analyze the repair of CPDs in the transcribed and non-transcribed strands of the *EMC7* gene. Briefly, 1 μg of genomic DNA was digested with the restriction enzyme NdeI to release the 10 kb *EMC7* fragment. To incise at CPD damage sites, half of each sample was treated with an excess amount of T4 endonuclease V, and the other half was vehicle-treated. DNA samples were loaded and resolved in alkaline agarose gels. Separated DNA in the gels was then transferred and fixed onto a positively charged nylon membrane. The transcribed and non-transcribed strands of the *EMC7* gene were hybridized with the complementary RNA probes labeled with [α-32P] UTP, respectively. Storage phosphor screens were exposed to the radioactive membranes and imaged using a phosphorimager system. The resulting bands were quantified using ImageJ.

### 2.11. Statistical Analyses

Data analyses were conducted using GraphPad Prism 10 software. Quantitative values for experimental data are presented as the mean ± standard deviation (SD). Statistical significance between groups was assessed using the Student’s *t*-test or one-way ANOVA, with *p*-values less than 0.05 considered statistically significant (* *p* < 0.05, ** *p* < 0.01, *** *p* < 0.001, **** *p* < 0.0001). Asterisks (*) and plus signs (^+^) were used to distinguish *p*-values for different comparisons within the data, enhancing clarity in interpretation. Additionally, box-and-whisker plots and violin plots were utilized to clarify the interpretation of data distribution.

## 3. Results

### 3.1. Senataxin Enhances Sensitivity to H_2_O_2_-Induced Cell Death

**Figure 1:**
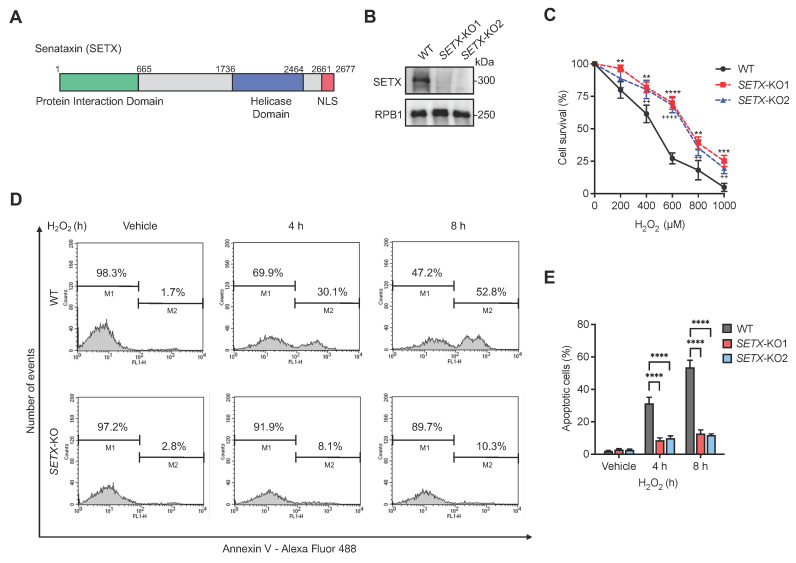
Senataxin sensitizes cells to apoptosis in response to H_2_O_2_. (**A**) Schematic diagram of the functional domain organization of the human senataxin protein, including the N-terminal protein interaction domain, the helicase domain, and the C-terminal nuclear localization signal (NLS). Numbers of amino acid residues are highlighted above. (**B**) Immunoblotting analysis of senataxin protein expression in WT and two candidates of *SETX* knockout R2F cell lines. RPB1 expression were detected to ensure equal loading. WT, wild-type; KO, knockout. (**C**) Cell viability assay showing the cell survival percentage of WT and *SETX* knockout cells treated with the indicated concentrations of H_2_O_2_ for 12 h. Statistical significance is denoted by asterisks (*) and pluses (+), where symbols represent *p*-values for different comparisons: * for the comparison of *SETX*-KO1 with the WT group (indicated by asterisks), and + for the comparison of *SETX*-KO2 with the WT group (indicated by pluses). (**D**) Representative flow cytometry analysis histograms from Annexin V/PI apoptosis assay, presenting the distribution of annexin V negative cells (M1) and annexin V positive cells (M2) in WT and *SETX* knockout cells after 4 h or 8 h incubation with 1mM H_2_O_2_. (**E**) Quantification of the apoptotic cells in the indicated cells treated as in (**D**). ** or ^++^ represents *p* < 0.01, *** represents *p* < 0.001, and **** or ^++++^ represents *p* < 0.0001.

As illustrated in Figure 1A, senataxin is a large protein comprising 2677 amino acid residues. Similar to its yeast homolog Sen1, it features an SF1B-type helicase domain (residues 1736–2464) that facilitates the unwinding of RNA–DNA hybrids [49]. Notably, missense mutations associated with AOA2 and ALS4 are predominantly located within this helicase domain. In addition to the helicase domain, senataxin possesses an N-terminal region (residues 1–665) that undergoes SUMOylation, in the form of SUMO1-mediated monoSUMOylation or SUMO2 and SUMO3-mediated polySUMOylation, depending on cellular context [50,51]. This modification facilitates protein–protein interactions with factors involved in RNA metabolism [52]. Furthermore, senataxin contains a C-terminal nuclear localization signal (NLS) (residues 2661–2677), which is crucial for its nuclear import and subsequent function [40].

To investigate the regulatory mechanisms and cellular effects of senataxin in response to various DNA damage-inducing agents, *SETX* gene knockout cell lines were established using the CRISPR/Cas9 genome editing technique. The successful knockout of the *SETX* gene was validated through Western blot analysis using a specific antibody against senataxin. Western blot results confirmed the complete knockout of *SETX* in two distinct CRISPR/Cas9 knockout clones, as evidenced by the absence of the senataxin protein compared to the wild-type (WT) control (Figure 1B).

Exogenous H_2_O_2_ is frequently utilized as a model oxidative stressor to study its impact on cellular and molecular mechanisms [53]. H_2_O_2_ induces several types of DNA damage, including the formation of oxidative base lesions, single-strand breaks, and DSBs, which collectively contribute to genomic instability and cellular dysfunction [53,54,55]. To assess the cellular effects of H_2_O_2_, the viability of WT and *SETX* knockout cells was evaluated 24 h post-treatment using trypan blue exclusion staining. Treatment with H_2_O_2_ at concentrations ranging from 200 to 1000 µM significantly decreased the viable population (trypan blue-negative) of WT cells in a dose-dependent manner, in contrast to the *SETX* knockout cells, which displayed a different survival profile under the same conditions (Figure 1C).

To further investigate the effects of H_2_O_2_-induced cell death, Annexin V-Alexa Fluor 488 and propidium iodide (PI) staining were employed to detect the rate of apoptosis. Annexin V has a high affinity for phosphatidylserine (PS), and its binding is critical for monitoring apoptotic cells, as PS externalization is a hallmark of early apoptosis [56]. Flow cytometric analysis was used to quantify the extent of apoptosis, with the peak area of the “M2” region representing the percentage of Annexin V-positive cells (apoptotic cells), and the “M1” region indicating the percentage of viable or necrotic cells. The results demonstrated a marked increase in the ratio of apoptotic cells in WT cells treated with H_2_O_2_ for 4 and 8 h compared to the *SETX* knockout cells, indicating that *SETX*-KO cells exhibit increased viability under oxidative stress compared to WT cells (Figure 1D,E).

### 3.2. Senataxin Is Required for Efficient Repair of Double-Strand Breaks Generated by I-PpoI

**Figure 2:**
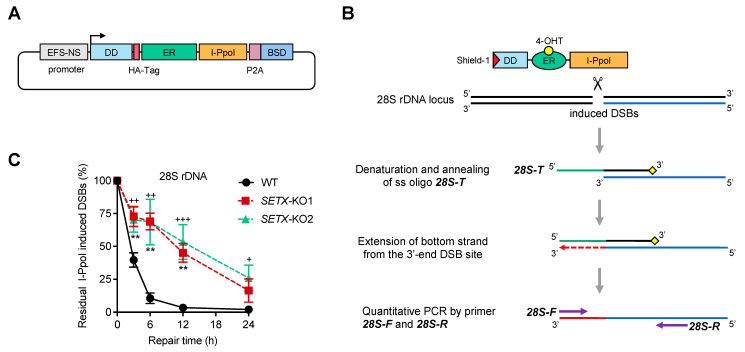
Senataxin has an essential role in DNA double-strand break repair. (**A**) Schematic illustration of the plasmid map of the recombinant construct pLenti-DD-HA-ER-I-PpoI. DD, destabilization domain; ER, estrogen receptor; BSD, blasticidin. (**B**) Schematic showing the procedure of the DSB repair assay. The recombinant I-PpoI was designed to fuse with DD and ER to control its expression, as the ligands Shield-1 and 4-OHT bind with DD and ER, respectively, leading to stabilized expression and nuclear localization of I-PpoI. DSBs at the 28S rDNA locus were conditionally generated by I-PpoI. A single-strand oligo 28s-T was added to anneal to the 3′ DSB region of the 28S rDNA after genomic DNA denaturation. Extension was performed from the 3′ end of the DSB, followed by qPCR using 28S-F and 28S-R primers to quantify the DSB-containing fragments. (**C**) Quantification of the percentage of residual I-PpoI-induced DSBs in the 28S rDNA locus of the indicated cells at different times of repair incubation, following treatment with 1 mM Shield-1 and 2 mM 4-OHT for 5 h to generate DSBs induced by I-PpoI. Statistical significance is denoted by asterisks (*) and pluses (+), where symbols represent *p*-values for different comparisons: * for the comparison of *SETX*-KO1 with the WT group (indicated by asterisks), and + for the comparison of *SETX*-KO2 with the WT group (indicated by pluses). ^+^ represents *p* < 0.05, ** or ^++^ represents *p* < 0.01, and ^+++^ represents *p* < 0.001.

To investigate the effects of DSB repair modulated by senataxin, we utilized an inducible I-PpoI platform. This system employs the fungal homing endonuclease I-PpoI, which targets a 15-base pair canonical site within the 28S ribosomal DNA (rDNA) coding region, thereby generating site-specific DSBs [57]. As shown in Figure 2A, stable expression of an HA-tagged I-PpoI fusion protein (I-PpoI-ER-DD-HA), incorporating both a destabilization domain (DD) and an estrogen receptor (ER) domain, was achieved using a lentiviral vector, followed by selection with blasticidin.

The Shield-1 compound is utilized to stabilize proteins tagged with the DD, preventing proteasomal degradation of the DD fusion protein of interest [58]. Concurrently, the anti-estrogen tamoxifen binds to the cytoplasmic estrogen receptor-fused protein, facilitating its translocation into the nucleus [59]. To achieve precise temporal control of I-PpoI induction, sequential treatments with the DD inhibitor Shield-1 (for 1 h) and 4-OHT (for 15 min) were employed, thereby initiating the DSB-inducing activity of I-PpoI within the nucleus.

To assess the level of DSBs over a time course, we measured DSBs at several time points, thereby enabling the illustration and comparison of DSB repair kinetics between different cell groups. As illustrated in Figure 2B, we established a robust strategy to achieve this objective. Specifically, synthesized single-stranded oligonucleotides (oligo) 28S-T, blocked at the 3′ end, were designed to anneal with the 3′ DSB site region of the 28S rDNA sequence following the denaturation of genomic DNA samples. DNA polymerase then performed 3′ end extension from the DSB site on the 28S rDNA strand, terminating extension at the complementary site of the 5′ end of oligo 28S-T. Quantitative PCR (qPCR) was subsequently conducted to quantify all extended 28S rDNA sequence copies using the primer pair 28S-F and 28S-R.

We aimed to compare the DSB repair kinetics between WT and *SETX* knockout cells. Following the induction of DSBs in the 28S rDNA region by I-PpoI, nuclear DNA was isolated at various repair incubation times, ranging from 0 to 24 h. The DSB levels were assessed using the method outlined in Figure 2B. The kinetics of DSB repair were plotted, revealing that *SETX* knockout cells exhibited a significantly higher proportion of residual I-PpoI-induced DSBs compared to WT cells (Figure 2C). These data suggest that senataxin is crucial for the efficient repair of DSBs within the DSB repair pathway.

### 3.3. Senataxin Attenuates ATM and ATR Signaling upon DNA Damage Induction

**Figure 3:**
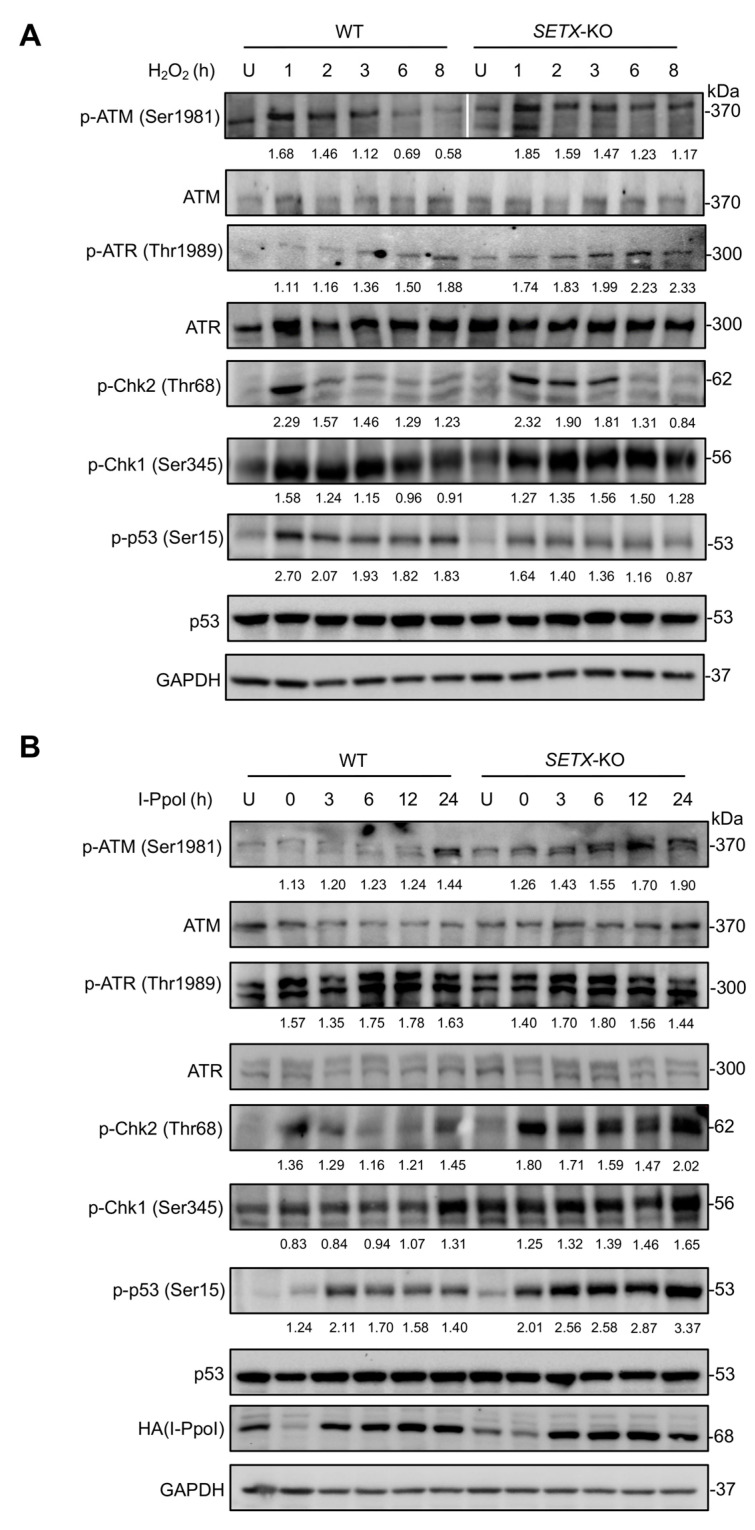
Loss of senataxin expression elevates and prolongs the activation of phosphorylation levels in the ATM-Chk2 and ATR-Chk1 signaling pathways in response to H_2_O_2_ treatment and I-PpoI induction. (**A**) Immunoblotting analysis of the activation of the ATM-Chk2-p53 and ATR-Chk1 signaling axes in WT and *SETX* knockout cells after the indicated incubation times of 300 μM H_2_O_2_ treatment. “U” refers to cells untreated with H_2_O_2_. GAPDH was used as a loading control. A thin white line has been added to the panel to indicate that the Western blot image was processed by cutting and combining lanes from the same original blot. (**B**) Immunoblotting analysis of the activation of the ATM-Chk2-p53 and ATR-Chk1 signaling axes in WT and *SETX* knockout I-PpoI-expressing cells after the indicated incubation times, within 24 h following I-PpoI induction for 5 h by 1 mM Shield-1 and 2 mM 4-OHT. “U” refers to cells untreated with Shield-1 and 4-OHT. GAPDH was used as a loading control. The relative band intensity was normalized and calculated by comparing the treated samples to the untreated (U) samples. The resulting values of this relative band intensity were labeled underneath the corresponding panels of the Western blot.

The observation that *SETX* knockout cells exhibit prolonged and elevated levels of phosphorylated ATM (p-ATM at Ser1981), ATR (p-ATR at Thr1989), Chk2 (p-Chk2 at Thr68), and Chk1 (p-Chk1 at Ser345) following H_2_O_2_ treatment and I-PpoI-induced DSBs suggests a critical role for senataxin in DNA damage resolution and DDR signaling (Figure 3). Senataxin is implicated in resolving R-loops, which, if unresolved, can lead to persistent DNA damage and continuous activation of DDR pathways. The elevated phosphorylation levels in knockout cells indicate an accumulation of unresolved DNA damage, likely due to inefficient repair of R-loops and DSBs, resulting in sustained ATM and ATR activation. This persistent activation leads to ongoing phosphorylation of downstream effectors Chk2 and Chk1. The prolonged DDR signaling reflects an impaired feedback mechanism, where the absence of senataxin hampers the resolution of DNA damage, leading to chronic activation of pATM, pATR, pChk1, and pChk2.

Phosphorylation at Ser15 enhances p53’s transcriptional activity, facilitating the expression of downstream genes involved in DNA repair, cell cycle arrest, and apoptosis [60]. Our findings underscore the pivotal role of senataxin in modulating p53 phosphorylation at Ser15 in response to oxidative stress induced by H_2_O_2_. In WT cells, p53 phosphorylation at Ser15 remains relatively higher post-H_2_O_2_ treatment, whereas *SETX* knockout cells exhibit consistently lower phosphorylation levels (Figure 3A). This indicates that senataxin is essential for maintaining p53 activation under persistent oxidative stress, thereby supporting its function in managing sustained DNA damage. The lower level of cell viability in WT cells treated with a similar concentration of H_2_O_2_ compared to *SETX* knockout cells (Figure 1C) further corroborates this, with the prolonged p53 phosphorylation in WT cells being associated with heightened p53 activity and the consequent induction of apoptosis in response to DNA damage. Conversely, the reduced phosphorylation of p53 in *SETX* knockout cells suggests a compromised apoptotic response, likely due to diminished p53 activity, which allows these cells to evade or delay apoptosis despite the presence of oxidative stress.

Following I-PpoI induction, WT cells exhibit lower levels of p53 phosphorylation at Ser15 (Figure 3B). This observation suggests that senataxin may enhance the efficiency of repairing I-PpoI-induced DSBs, thereby reducing the need for sustained p53 activation. The reduced p53 phosphorylation in WT cells could indicate a more effective or expedited resolution of DSBs, leading to diminished activation of the DDR. In contrast, *SETX* knockout cells display significantly higher and prolonged levels of p53 phosphorylation at Ser15 following I-PpoI induction (Figure 3B). This prolonged phosphorylation suggests that, in the absence of senataxin, cells encounter increased difficulty in repairing DSBs, resulting in sustained p53 activation. The persistent and elevated p53 phosphorylation in *SETX* knockout cells likely reflects an unresolved DDR, underscoring senataxin’s role in facilitating or coordinating DSB repair. The lack of apoptosis in both WT and *SETX* knockout cells, treated with Shield-1 and 4-OHT to activate I-PpoI, despite variations in p53 phosphorylation levels, suggests that the extent of damage caused by I-PpoI may be insufficient to activate apoptotic pathways. Cells might perceive the damage as manageable, leading to the activation of DNA repair mechanisms rather than apoptosis. This indicates that, in this context, p53 activation is primarily involved in promoting DNA repair and cell survival rather than apoptosis. The cells likely utilize p53 to enforce cell cycle arrest or activate repair pathways, thereby facilitating survival despite DSBs induced by I-PpoI. Thus, the differing levels of p53 phosphorylation reflect variations in the DDR but do not necessarily correlate with apoptotic outcomes.

In summary, the differential outcomes in p53 phosphorylation observed in these experimental contexts highlight the distinct roles of senataxin in managing oxidative stress versus DSB repair. Under oxidative stress, senataxin is critical for maintaining p53 activation and facilitating apoptosis. Conversely, in the context of DSBs, senataxin appears to be involved in the efficient resolution of DNA damage, thereby diminishing the necessity for prolonged p53 activation.

### 3.4. Senataxin Is Involved in Suppressing DNA Damage-Induced Senescence

Senescence is closely associated with the ATM/ATR DDR signaling pathways, which are activated in response to DSBs to maintain genomic integrity. Persistent activation of these pathways due to unresolved DSBs can lead to cellular senescence [61,62]. Given that senataxin is essential for DSB repair through ATM/ATR signaling pathways, we aimed to elucidate the role of senataxin in determining cell fate regarding senescence.

**Figure 4:**
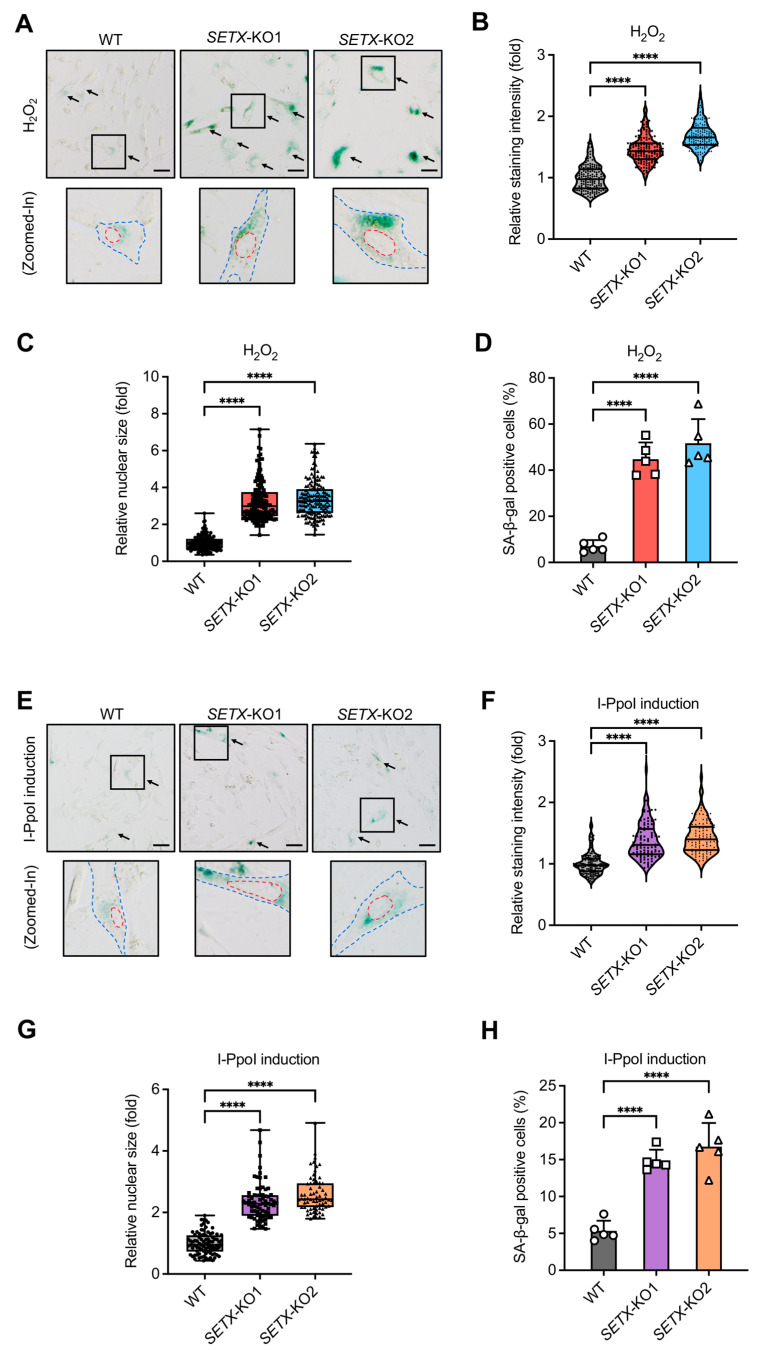
Senataxin deficiency triggers the increase of cellular senescence caused by H_2_O_2_ and I-PpoI. (**A**) Representative microscopic images of cells induced to senescence by 200 μM H_2_O_2_ over a 4-day period, detected using the SA-β-gal assay. One of the most typical senescent cells from each sample was selected with a square, of which the area was zoomed in, and the nuclei region was outlined with a red dotted line, while the entire cell was outlined with a blue dotted line. Black arrows point to the typical senescent cells. Scale bar, 20 μm. (**B**–**D**) Quantification of relative staining intensity ((**B**), fold), relative nuclear size ((**C**), fold), and percentage of SA-β-gal positive cells in the treated cells shown in (**D**). WT was set as control of 1 in (**B**,**C**). (**E**) Representative microscopic images of I-PpoI-induced senescent cells detected by the SA-β-gal assay, following treatment with 1 mM Shield-1 and 2 mM 4-OHT for 4 days. As in (**A**), one of the most typical senescent cells from each sample was selected with a square, of which the area was zoomed in, with the nuclei region outlined in a red dotted line and the entire cell outlined with a blue dotted line. Black arrows point to the typical senescent cells. Scale bar, 20 μm. (**F**–**H**) Quantification of relative staining intensity ((**F**), fold), relative nuclear size ((**G**), fold), and percentage of SA-β-gal positive cells in the treated cells shown in (**H**). WT was set as the control of 1 in (**B**,**C**,**F**,**G**). **** represents *p* < 0.0001.

One of the most commonly used markers of senescence is increased activity of β-galactosidase, termed SA-β-gal [63]. As models of senescence, we utilized H_2_O_2_-induced senescence (DIS), induced by treating cells with H_2_O_2_, and I-PpoI-induced senescence (IRIS), induced by treating cells with Shield-1 and 4-OHT. The SA-β-gal assay confirmed cellular senescence in each treatment condition. We detected SA-β-gal activity and observed that positive staining in WT cells was rare, whereas the frequency of SA-β-gal-positive *SETX* knockout cells increased significantly upon H_2_O_2_ and I-PpoI treatment. Senescent cells exhibited a flattened and expanded cytoplasmic morphology with increased SA-β-gal activity (Figure 4A,E). Statistical analysis revealed that the percentage of SA-β-gal-positive cells in *SETX* knockout cells was significantly higher than in control cells (Figure 4D,H). Additionally, all senescent cells in *SETX* knockout samples had a significantly larger nuclear area compared to control cells (Figure 4C,G). The comparison of the relative intensity of β-galactosidase staining between samples was consistent with these observations (Figure 4B,F).

### 3.5. Depletion of Senataxin Promotes UVC-Induced Apoptotic Cell Death

**Figure 5:**
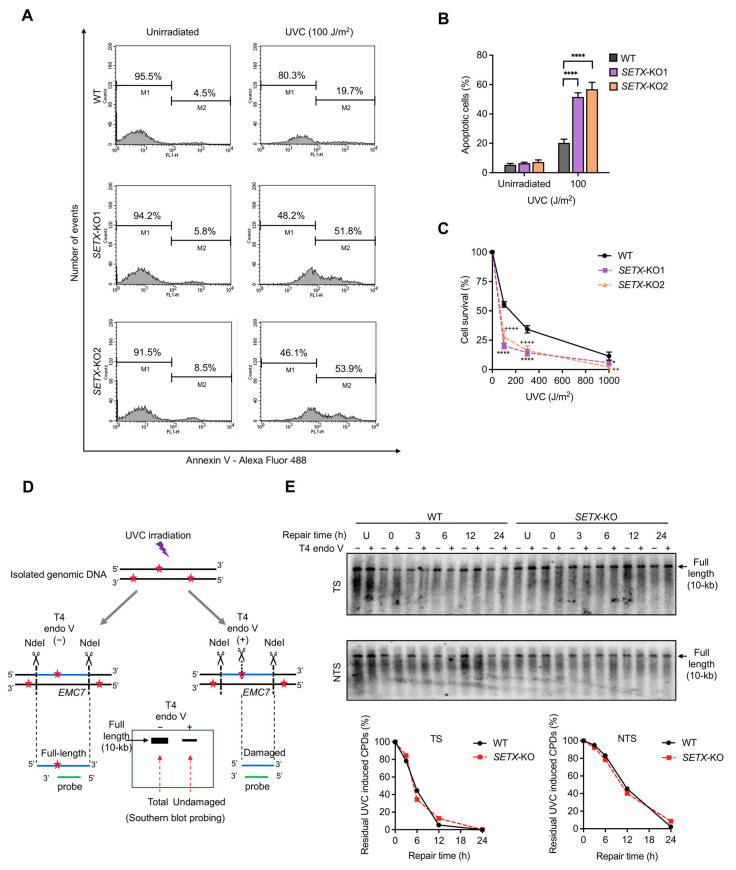
Senataxin is critical for maintaining cell viability following UV irradiation. (**A**) Representative flow cytometry analysis histograms from the Annexin V/PI apoptosis assay, presenting the distribution of annexin V negative cells (M1) and annexin V positive cells (M2) in WT and *SETX* knockout cells after a 24 h repair period following 100 J/m² UVC irradiation. (**B**) Quantification of the apoptotic cells in the indicated cells treated as in (**A**). (**C**) Cell viability assay showing the cell survival percentage of WT and *SETX* knockout cells after a 48 h repair period following the indicated doses of UVC irradiation. Statistical significance is denoted by asterisks (*) and pluses (+), where symbols represent *p*-values for different comparisons: * for the comparison of *SETX*-KO1 with the WT group (indicated by asterisks), and + for the comparison of *SETX*-KO2 with the WT group (indicated by pluses). (**D**) Schematic illustration of the experimental setup to investigate potential differences in UVC-induced CPD repair rates between WT and *SETX* knockout cells. Cells were irradiated with 25 J/m² UVC to induce CPD damage and incubated for various repair durations. Total genomic DNA was isolated and digested with NdeI to release a 10 kb fragment of the *EMC7* gene. Half of the DNA samples were treated with T4 endonuclease (endo) V, which cleaves DNA strands containing CPD lesions, while the other half remained untreated. All samples underwent Southern blot analysis, with a probe targeting the 10 kb fragment of the *EMC7* gene. In the Southern blot, the 10 kb full-length band intensity was quantified. For the T4 endo V-untreated samples, the 10 kb band represents the total amount of both damaged and undamaged fragments, whereas for the T4 endo V-treated samples, the 10 kb band represents only the undamaged fragments (as the damaged fragments are shorter than 10 kb). The residual CPD percentage was calculated based on the band intensities of the full-length 10 kb *EMC7* gene fragments from both treated and untreated samples. CPD lesions are indicated by red stars, while the 10 kb *EMC7* fragment targeted for repair analysis is depicted by a blue line, with the hybridization probe shown as a green line. (**E**) Southern blot analysis showing UVC-induced CPD repair at different repair incubation times in WT and *SETX* knockout cells on both the transcribed strand (TS) and the non-transcribed strand (NTS). The setup follows the schematic shown in (**D**). Unirradiated cell samples (U) served as negative controls. The arrow-marked bands at the top correspond to the 10 kb full-length restriction fragment of the *EMC7* gene. The quantification of the percentage of residual CPDs across various repair time points is presented for both strands, comparing WT and *SETX* knockout cells. * represents *p* < 0.05, ^++^ represents *p* < 0.01, and **** or ^++++^ represents *p* < 0.0001.

NER is a common and essential DNA repair pathway in human cells, responsible for removing bulky DNA adducts such as UV radiation-induced CPDs and chemical adducts [64]. We aimed to investigate the role of senataxin in response to CPD damage. To this end, annexin V/PI staining assays were performed to reveal the ratio of apoptotic cells (denoted as the “M2” region) following varying dosages of UVC irradiation. The amount of Alexa Fluor 488-labeled annexin V bound to cells directly correlates with apoptosis levels. Therefore, cells undergoing higher apoptosis exhibit greater fluorescence intensity, resulting in the shift of the M1 peak (annexin V-negative) toward the right on the x-axis of FL1-H green fluorescence intensity, as these cells transition into the M2 peak (annexin V-positive) (Figure 5A). Statistical analysis demonstrated that *SETX* knockout cells exhibited a significantly higher percentage of apoptotic cells compared to WT cells in response to UVC irradiation (Figure 5A,B). Corroborating these findings, survival curve plots from trypan blue staining assays indicated that the cell survival rate of *SETX* knockout cells was markedly lower than that of WT cells after exposure to different dosages of UVC irradiation, in a dose-dependent manner (Figure 5C). These results suggest that senataxin plays a critical role in protecting cells from apoptosis and maintaining cell viability following UVC-induced CPD damage.

To investigate potential differences in the repair rates of UVC-induced CPD DNA damage between different cell groups, we analyzed strand-specific repair within a 10 kb NdeI restriction fragment encompassing the *EMC7* gene region. The experimental setup is illustrated in the schematic of Figure 5D. Cells were first irradiated with 25 J/m^2^ UVC and then incubated for varying repair durations. Subsequently, total genomic DNA was isolated after each repair incubation period. The isolated DNA samples were digested with the NdeI restriction enzyme, releasing a 10 kb full-length fragment located within the *EMC7* gene. Half of the DNA samples were treated with T4 endonuclease V, which recognizes and cleaves DNA strands containing CPD lesions, while the other half remained untreated. All DNA samples were then subjected to Southern blot analysis: they were first separated on alkaline agarose gels, transferred to membranes, and hybridized with radiolabeled probes targeting the restriction fragment of the *EMC7* gene.

The autoradiograms in Figure 5E display bands at the 10 kb position, which represent the full-length *EMC7* gene fragments detected by the probe, as illustrated in Figure 5D. For T4 endonuclease V-untreated samples (−), the 10 kb band intensity reflects the total amount of the full-length *EMC7* gene fragments, including both damaged and undamaged fragments. In contrast, for T4 endonuclease V-treated samples (+), the 10 kb band intensity indicates the amount of undamaged *EMC7* gene fragments, as the damaged fragments are cleaved by T4 endonuclease V, resulting in shorter fragments that are not detected at the 10 kb position. Residual UVC-induced CPDs (%) of each repair time (h) was calculated using the band intensity at the full-length 10 kb location with the formula:

(Total band intensity − Undamaged band intensity)/Total band intensity × 100%

The quantification analyses of the autoradiograms demonstrated no significant differences in the kinetics and extent of CPD repair between WT and *SETX* knockout cells on both strands of the *EMC7* gene (Figure 5E). These findings suggest that senataxin does not play a significant role in the NER of CPD-induced DNA damage.

## 4. Discussion

Our study sheds light on the multifaceted role of senataxin in maintaining genomic integrity, particularly through its involvement in the repair of DSBs and the suppression of cellular senescence. The findings presented herein provide compelling evidence that senataxin plays a critical role in orchestrating cellular responses to oxidative stress and genotoxic insults, primarily by modulating key DDR pathways. The observed heightened and prolonged activation of DDR components such as ATM, ATR, Chk2, and Chk1 in senataxin-deficient cells underscores the protein’s essential function in promoting efficient DSB repair. A novel finding in this study is that the extended DDR activation in *SETX* knockout cells, although protective initially, results in chronic cellular stress, driving increased senescence rather than apoptosis. This suggests that senataxin’s regulation of DDR signaling not only prevents genomic instability but also influences the cellular aging process by modulating the balance between apoptosis and senescence. Our findings highlight senataxin as a key regulator in mitigating senescence-associated dysfunctions, offering potential therapeutic avenues for age-related diseases linked to impaired DNA repair.

The differential response of WT and *SETX* knockout cells to H_2_O_2_-induced oxidative stress further elucidates senataxin’s role in apoptosis regulation. Our data demonstrate that senataxin-deficient cells exhibit a lower rate of apoptosis compared to WT cells, despite prolonged DDR activation. This suggests that senataxin promotes apoptosis over senescence in response to oxidative stress, potentially acting as a modulator of cell fate decisions following DNA damage. The delay in apoptosis observed in knockout cells may represent an adaptive response, allowing these cells to survive longer under conditions of persistent DNA damage, albeit with increased genomic instability. Prolonged DDR activation in response to H_2_O_2_ can drive cells toward senescence, with ATM and p53 playing critical roles in fine-tuning the balance between senescence and apoptosis [65,66]. However, the cross-regulation between apoptosis and cellular senescence, particularly the involvement of senataxin in maintaining this balance, remains poorly understood, necessitating further studies to clarify their interrelationships. Additionally, the observed apoptosis may not solely result from DNA DSBs but could also be influenced by other types of damage induced by H_2_O_2_ exposure. Therefore, future studies using reagents that specifically induce DSBs could provide deeper insights into the role of senataxin in apoptosis in response to DSBs.

The previous study [38] reported reduced viability of *SETX*-mutated cells exposed to H_2_O_2_ compared to normal controls, which contrasts with our findings of increased viability in senataxin knockout cells under H_2_O_2_ treatment. This discrepancy likely arises from the partial loss of senataxin expression in the patient-derived cells used in their study. The cells used in their study retained residual senataxin due to partial exon deletions or splicing defects, which could have contributed to their altered sensitivity to oxidative stress. Specifically, the *SETX* mutants used in this study only lost a section of the helicase domain, which may partially affect senataxin’s function. In contrast, our complete knockout model fully eliminated senataxin expression, leading to increased viability and reduced apoptosis. This underscores the significance of complete knockout models in accurately elucidating gene function. Additionally, the treatment conditions differ significantly between studies. The previous study employed a 30 min treatment with 0.05–0.2 mM H_2_O_2_, with viability measured after 4 days. In contrast, our study used higher H_2_O_2_ concentrations (0.2–1 mM) for a 12 h exposure, with viability measured 24 h post-treatment. These differences in treatment duration and concentration may explain the contrasting results, emphasizing the impact of experimental conditions in assessing cellular responses to oxidative stress. Differences in genetic background or compensatory mechanisms between the cell lines may also contribute. The previous study showed that DDX47 complements SETX function, suppressing DNA–RNA hybrid accumulation in *SETX*-depleted cells [67,68], suggesting that potential compensation in the absence of *SETX* might explain the discrepancy in results between different cell lines. Further rescue experiments are needed in future studies to confirm that the observed phenotypes are specifically attributable to *SETX* knockout alone, as the CRISPR/Cas9 system employed may have introduced potential off-target effects.

Our investigation into the role of senataxin in the repair of I-PpoI-induced DSBs provides additional insights into the protein’s function in preserving genomic stability. The significantly slower DSB repair kinetics observed in *SETX* knockout cells compared to WT cells highlights the importance of senataxin in facilitating efficient DSB repair, particularly within the homologous recombination pathway. This is consistent with a previous finding that AOA2 cells exhibited significantly reduced rejoining of H_2_O_2_-induced DSBs compared to controls, and that this impairment in DSB repair was reversed by full-length *SETX* cDNA, highlighting senataxin’s essential role in DSB repair [38]. The impaired DSB repair in the absence of senataxin likely leads to the persistence of DNA damage, which, as demonstrated in our study, triggers sustained ATM and ATR signaling. This chronic activation of DDR pathways not only promotes cellular senescence but also underscores the potential of senataxin as a therapeutic target for mitigating premature aging and related disorders by enhancing DNA repair capacity. These findings align with previous studies linking chronic DDR activation and the subsequent promotion of cellular senescence, contributing to the understanding of how senataxin deficiency may accelerate aging and age-related pathologies [69,70].

Finally, our study explored the role of senataxin in response to UVC-induced DNA damage, specifically in the context of NER. Contrary to its pronounced effect on DSB repair, our findings suggest that senataxin does not significantly influence the repair of UVC-induced CPDs. This observation delineates the specificity of senataxin’s function within distinct DNA repair pathways, focusing on the resolution of R-loops and DSBs. Under UVC radiation, transcriptional stalling at CPD damage sites intensifies R-loop formation by hindering normal transcription elongation and increasing RNA–DNA hybridization [71]. RNase H helps resolve these R-loops by degrading the RNA component [72]. Additionally, senataxin plays a crucial role in unwinding RNA–DNA hybrids, preventing genomic instability [43]. Recent findings also reveal that USP11 regulates R-loop dynamics by deubiquitinating and stabilizing senataxin [73]. DHX9 and DDX19 are among the first DEAH-box and DEAD-box helicases identified for their roles in resolving R-loops [67]. DHX9 suppresses R-loops and regulates transcriptional termination through ATR-induced phosphorylation in response to DNA damage, relying on its interaction with RPA [74,75]. DDX19 is phosphorylated by Chk1 during replication stress or DNA damage, activating the ATR-Chk1 pathway and promoting its relocalization to the nucleus to resolve R-loops and maintain genomic integrity [76]. Moreover, DEAD-box helicases like DDX17 and DDX41, which function similarly to SETX, aid in mitigating R-loop-induced replication stress [77,78]. Although NER efficiently repairs CPDs, *SETX* knockout cells experience heightened replication stress due to unresolved R-loops, leading to increased replication fork instability, collapse, and apoptosis [79].

Future research should explore the broader implications of senataxin’s role in DNA repair and cellular senescence, particularly focusing on its potential as a therapeutic target for age-related diseases and neurodegenerative disorders. While this study highlights senataxin’s involvement in repairing DNA DSBs and modulating cellular responses to oxidative stress, further investigations are needed to elucidate the molecular mechanisms underlying its interaction with other DNA repair proteins and pathways. Moreover, examining how senataxin deficiency impacts long-term genomic stability and contributes to premature aging in various cell types could provide critical insights into age-associated pathologies. Additionally, research into small molecules or gene therapy approaches that can enhance or mimic senataxin function might offer novel strategies for alleviating genomic instability and preventing the onset of senescence-related diseases. The role of senataxin, as highlighted in previous studies, suggests that targeting senataxin pathways could be beneficial in addressing genomic instability in neurodegenerative diseases [80,81].

In summary, our study provides novel findings that senataxin deficiency prolongs DDR activation, shifting cells from apoptosis to senescence. This chronic stress response accelerates aging, emphasizing senataxin’s role in maintaining genomic stability and offering potential therapeutic insights for age-related diseases involving impaired DNA repair and senescence.

## Data Availability

The original contributions presented in the study are included in the article. The data supporting this study’s findings are available upon request from the corresponding author.
